# Expression of cyanobacterial FBP/SBPase in soybean prevents yield depression under future climate conditions

**DOI:** 10.1093/jxb/erw435

**Published:** 2016-12-12

**Authors:** Iris H. Köhler, Ursula M. Ruiz-Vera, Andy VanLoocke, Michell L. Thomey, Tom Clemente, Stephen P. Long, Donald R. Ort, Carl J. Bernacchi

**Affiliations:** 1 Global Change and Photosynthesis Research Unit, Agricultural Research Service, United States Department of Agriculture, Urbana, IL 61801, USA; 2 Carl R. Woese Institute for Genomic Biology, University of Illinois at Urbana-Champaign, Urbana, IL 61801, USA; 3 Department of Agronomy, Iowa State University, Ames, IA 50011, USA; 4 Center for Plant Science Innovation, University of Nebraska-Lincoln, Lincoln, NE 68588, USA; 5 Lancaster Environment Centre, University of Lancaster, Lancaster LA1 4YQ, UK; 6 Departments of Plant Biology and Crop Sciences, University of Illinois at Urbana-Champaign, Urbana, IL 61801, USA

**Keywords:** Elevated CO_2_, elevated temperature, free air CO_2_ enrichment, *Glycine max*, sedoheptulose-1,7-bisphosphatase, Soy-T-FACE.

## Abstract

Predictions suggest that current crop production needs to double by 2050 to meet global food and energy demands. Based on theory and experimental studies, overexpression of the photosynthetic enzyme sedoheptulose-1,7-bisphosphatase (SBPase) is expected to enhance C_3_ crop photosynthesis and yields. Here we test how expression of the cyanobacterial, bifunctional fructose-1,6/sedoheptulose-1,7-bisphosphatase (FBP/SBPase) affects carbon assimilation and seed yield (SY) in a major crop (soybean, *Glycine max*). For three growing seasons, wild-type (WT) and FBP/SBPase-expressing (FS) plants were grown in the field under ambient (400 μmol mol^−1^) and elevated (600 μmol mol^−1^) CO_2_ concentrations [CO_2_] and under ambient and elevated temperatures (+2.7 °C during daytime, +3.4 °C at night) at the SoyFACE research site. Across treatments, FS plants had significantly higher carbon assimilation (4–14%), *V*_c,max_ (5–8%), and *J*_max_ (4–8%). Under ambient [CO_2_], elevated temperature led to significant reductions of SY of both genotypes by 19–31%. However, under elevated [CO_2_] and elevated temperature, FS plants maintained SY levels, while the WT showed significant reductions between 11% and 22% compared with plants under elevated [CO_2_] alone. These results show that the manipulation of the photosynthetic carbon reduction cycle can mitigate the effects of future high CO_2_ and high temperature environments on soybean yield.

## Highlight

Expression of cyanobacterial FBP/SBPase in soybean prevents yield depression in a free air CO_2_ enrichment experiment under simulated future climate conditions with combination of elevated CO_2_ and elevated temperature.

## Introduction

Crop productivity may have to increase by 60**–**110% over 2005 levels by 2050 (Tilman *et al.*, 2011; Alexandratos and Bruinsma, 2012; OECD/FAO, 2012) to meet growing global food and energy demand. At the same time, atmospheric CO_2_ concentrations [CO_2_] are predicted to reach 550 μmol mol^−1^ by 2050 (IPCC, 2013) and this increase will be accompanied by an increase in terrestrial surface air temperatures of between 1 °C and 6 °C relative to 1961–1990, depending on geographic location (Rowlands *et al.*, 2012). Thus, approaches to improve crop yields need to take global climate change and the predicted future environmental conditions into account.

An apparent major opportunity to increase crop yields in the future is via improving photosynthetic efficiency. Various approaches to achieve this goal have been proposed (Zhu *et al.*, 2010; Blankenship *et al.*, 2011; Raines, 2011; Ort *et al.*, 2015). Particularly promising for future climatic and atmospheric conditions is increasing the rate of ribulose-1,5-bisphosphate (RuBP) regeneration (Raines, 2006, 2011). Under current atmospheric conditions, C_3_ photosynthesis (*A*) is mostly limited by the capacity for carboxylation by Rubisco, while under future elevated [CO_2_] and higher temperatures, the leaf photosynthesis model of carbon uptake and assimilation (Farquhar *et al.*, 1980; von Caemmerer and Farquhar, 1981; von Caemmerer, 2000) predicts that limitation will shift towards the regeneration capacity of RuBP (Long *et al.*, 2004). In the absence of other changes, rising temperature would increase the activity of Rubisco, but also lower its specificity for CO_2_ relative to O_2_. On balance, however, this will narrow the range of intercellular [CO_2_] under which Rubisco is limiting, and lower the [CO_2_] at which RuBP regeneration becomes limiting. In theory, the advantage of an increased capacity for RuBP regeneration would therefore be greatest under conditions of combined elevation of temperature and [CO_2_]. Ideally, future crops will be co-adapted to conditions of elevated [CO_2_] and higher temperatures. Thus, theoretically, enhancing RuBP regeneration capacity would be an effective strategy to adapt *A* to the higher atmospheric [CO_2_] and temperatures expected as climate change progresses.

The rate of RuBP regeneration can be limited by electron transport rates or by key enzymes in the photosynthetic carbon reduction (PCR) cycle. Using a complete dynamic model of photosynthetic carbon metabolism coupled to an evolutionary algorithm, Zhu *et al.* (2007) showed that optimizing the distribution of resources among PCR cycle enzymes is predicted to increase photosynthetic rates in CO_2_-enriched atmospheres. They predicted that levels of sedoheptulose-1,7-bisphosphatase (SBPase; EC 3.1.3.37) were suboptimal for maximizing *A* in the current atmosphere and even more suboptimal in future elevated CO_2_ atmospheres. SBPase is unique to the PCR cycle and catalyzes the dephosphorylation of sedoheptulose-1,7-bisphosphate to sedoheptulose-7-phosphate at the branch point of RuBP regeneration and carbon export (Raines *et al.*, 2000). In theory, an increase in the RuBP regeneration capacity through increased activity of SBPase, assuming no ATP or NADPH limitation, would lead to higher *A* under conditions where RuBP regeneration becomes limiting, namely under high [CO_2_] and higher temperatures, as noted earlier.

Theory and model predictions are also supported by experimental evidence. Flux control analysis with SBPase antisense tobacco has shown that the enzyme can exert a strong control on *A* (Harrison *et al.*, 1998; Raines, 2003), and overexpression of SBPase in tobacco increased both *A* and biomass (Lefebvre *et al.*, 2005; Rosenthal *et al.*, 2011; Simkin *et al.*, 2015). Increases in *A* and growth were also observed when a cyanobacterial bifunctional form of the enzyme (fructose-1,6/sedoheptulose-1,7-bisphosphatase, FBP/SBPase) was expressed in tobacco (Miyagawa *et al.*, 2001). The bifunctional FBP/SBPase has the same function as the two separate enzymes in higher plants, but has the advantage of being less prone to transgene silencing (Miyagawa *et al.*, 2001).

However, species and growth conditions may impact the above responses. Rice overexpressing SBPase did not show an enhancement of *A* or growth, but maintained *A* and growth rate under salt (Feng *et al.*, 2007*a*) and heat (Feng *et al.*, 2007*b*) stress conditions, relative to the wild-type (WT) plants. Lefebvre *et al.* (2005) observed that tobacco plants overexpressing SBPase did not show increases in *A* or yield under low light levels and short-day conditions. Differences between WT and SBPase-overexpressing plants were also dependent on the developmental stage (Lefebvre *et al.*, 2005; Rosenthal *et al.*, 2011). Overall, these results suggest that overexpression of SBPase or the expression of a bifunctional FBP/SBPase can lead to immediate improvements in *A* and plant growth; however, the response may vary among species and environmental conditions. None of the studies tested the effect of combined elevation of temperature and [CO_2_], where an increased capacity for RuBP regeneration would be most beneficial, as explained above. Thus, it is as yet unclear if overexpression of SBPase could be a potential means of improving *A* and yield in crop species in the field and under predicted future growth conditions.

The interaction between increased SBPase activity and future growth conditions was tested in the present study using soybean (*Glycine max*), a major crop species and the most widely grown legume worldwide (Ainsworth *et al.*, 2012). Soybean production increased from 27 Mt in 1961 to 308 Mt in 2014 (FAOSTAT 2016, http://faostat3.fao.org/, last accessed 19 October 2016) and is predicted to increase to 371 Mt by 2030 (Masuda amd Goldsmith, 2009). Soybean production since the year 2000 has nearly doubled, but this has been mainly achieved through increased land area for soybean cultivation (+60%), while soybean yields increased by only 19% (FAOSTAT 2016, http://faostat3.fao.org/, last accessed 19 October 2016). Given the limited availability of farmland and the negative environmental impacts of conversion of natural ecosystems, increases in soybean yield per area are a necessity for future sustainable increases in production. Experiments with soybean under elevated [CO_2_] and elevated temperatures indicate that CO_2_ alone will not increase yields in the future. In a field study, Ruiz-Vera *et al.* (2013) observed that in a warmer than average year for the Midwestern USA, elevated [CO_2_] was not able to mitigate the yield losses attributed to warming. This observation further emphasizes the importance of adapting crops to warmer and CO_2_-enriched environmental conditions.

To investigate if increased SBPase activity will increase yield in soybean under future climate, we grew WT and FBP/SBPase-expressing (FS) plants side by side under ambient and elevated [CO_2_] (400 μmol mol^−1^ and 600 μmol mol^−1^, respectively) combined with canopy heating of +2.7 °C during the day and +3.4 °C at night. The full factorial experiment was replicated over three growing seasons (2013–2015) at the SoyFACE research facility in central Illinois, USA. We hypothesized that expression of the cyanobacterial FBP/SBPase bifunctional enzyme would increase RuBP regeneration capacity (*J*_max_) and thus *A* under environmental conditions that favor RuBP regeneration-limited *A* (high light, elevated [CO_2_], warmer temperatures), and that the increases in *A* would be reflected in higher yields.

## Materials and methods

### Site description and experimental set-up

Soybean [*Glycine max* (L.) Merr. cv. ‘Thorne’] WT and FS plants derived from the same cultivar were grown in a complete block design (*n*=4) in the Soybean Temperature by Free Air CO_2_ Enrichment (Soy-T-FACE) experiment at the SoyFACE field site near Urbana-Champaign, IL, USA (40°2'30.49''N, 88°13'58.80''W, 230 m above sea level) during the 2013, 2014, and 2015 growing seasons. The experiment consisted of four blocks, each containing one ambient and one elevated [CO_2_] plot. Within each plot was nested an unheated and a heated subplot. Each subplot was further divided with plantings of the WT and FS lines. Seeds were planted by hand at 5 cm intervals in 38 cm rows. Eight 11 m long rows, four of WT and four of FS, were planted next to each other in each of the eight plots. Planting and harvest dates are given in Table 1. The ambient [CO_2_] plots were at ~400 μmol mol^−1^ and the elevated plots were fumigated to ~600 μmol mol^−1^ using free air CO_2_ enrichment (FACE) technology (Miglietta *et al.*, 2001). The heated subplots were each equipped with an infrared heater array, as described in detail previously (Ruiz-Vera *et al.*, 2013), installed at 1.0–.2 m above the canopy on a telescopic mast system (Ruiz-Vera *et al.*, 2015). Using a proportional–integral–derivative (PID) feedback control system, we warmed the crop canopy to a target elevation of +3.5 °C above that of the canopy temperature in the unheated subplot. The target temperature increase was based on the low-response model predictions for surface temperature in the Midwest in 2050 (Rowlands *et al.*, 2012).

During the day (6:00 h to 18:00 h), and with rainy days excluded, mean temperature differences between the subplots were between 0.5 °C and 1.0 °C lower than the target set point (Table 1), resulting in an average temperature increase of +2.7 °C. During the night, the average temperature difference was +3.4 °C. The heated subplot diameter was 3.5 m, resulting in an effective heated subplot area of 9.6 m^2^. Canopy temperature in each subplot was measured by infra-red radiometers (SI-111, Apogee Instruments, Logan, UT, USA) connected to data-loggers (CR1000 Micrologger, Campbell Scientific, Logan, UT, USA). Canopy temperature measurements were collected every 5 s to control the heater output and 10 min mean values were stored. The actual mean season canopy temperatures for the four treatments in the three seasons are given in Table 1. The canopy was heated continuously day and night from the VC (cotyledons expanded, Ritchie *et al.*, 1993) growth stage until harvest. Heaters were programmed to reduce energy output during precipitation events using a rain detector (Model 260-2590 Precipitation Detector, Nova Lynx Corporation, Grass Valley, CA, USA), as the target temperature difference cannot be maintained during precipitation. The treatments are hereafter referred to as ‘*Ac*’ (ambient [CO_2_], control temperature), ‘*Ah*’ (ambient [CO_2_]+heated), ‘*Ec*’ (elevated [CO_2_], control temperature), and ‘*Eh*’ (elevated [CO_2_]+heated).

**Table 1.** Planting and harvest dates and climatic parameters in the study years Canopy temperature (in °C) is averaged for the time period from canopy closure (V5, fifth node stage) to R7 (beginning of maturity) developmental stages (Ritchie *et al.*, 1993) on the control (*c*) and heated (*h*) plots under ambient (400 μmol m^−2^ s^−1^) and elevated (600 μmol m^−2^ s^−1^) [CO_2_] with periods of rain excluded for the calculations. ΔCanopy temperature (in °C) is the difference between heated and control plots within each CO_2_ treatment. Day is averaged from 6:00 h to 18:00 h and night from 18:00 h to 6:00 h.

Year	2013	2014	2015
ø Annual air temperature (°C)	10.7	9.8	11.6
ø Air temperature June–October (°C)	20.1	19.5	20.4
Annual sum precipitation (mm)	845	1012	1114
Sum precipitation June–October (mm)	363	525	536
ø Canopy temperature (°C) *c*/*h*			
Day: ambient [CO_2_]	25.7/28.6	23.3/26.2	24.7/27.2
Day: elevated [CO_2_]	26.6/29.3	23.7/26.3	25.0/27.6
Night: ambient [CO_2_]	17.4/20.8	15.4/18.9	17.3/20.7
Night: elevated [CO_2_]	17.7/21.1	15.7/19.1	17.1/20.5
ø ΔCanopy temperature (°C) ±SD			
Day: ambient [CO_2_]	3.0 ± 1.0	2.8 ± 1.0	2.5 ± 1.0
Day: elevated [CO_2_]	2.8 ± 0.8	2.7 ± 1.0	2.6 ± 1.1
Night: ambient [CO_2_]	3.4 ± 0.7	3.4 ± 0.4	3.4 ± 0.4
Night: elevated [CO_2_]	3.4 ± 0.6	3.4 ± 0.4	3.4 ± 0.5
Planting date (DOY)	158	169/170	156
Canopy closure V5 (DOY)	191	206	190
Beginning maturity R7 (DOY)	261	265	260
Harvest date, DOY (treatment)	275	281 (*Ah*, *Eh*) 292 (*Ac*, *Ec*)	274 (*Ac*) 275 (*Ec*, *Eh*) 279 (*Ec*, *Eh*) 280 (*Ah*, *Ec*, *Eh*)

Ø, average; DOY, day of year.

### Weather conditions

**Fig. 1.**
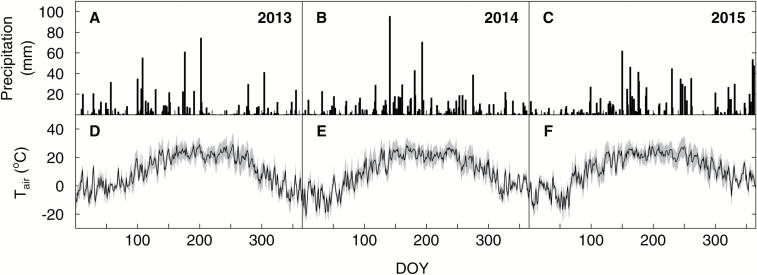
Precipitation and air temperature as recorded at Willard Airport Weather Station (http://mrcc.isws.illinois.edu/CLIMATE/, last accessed 2 August 2016) near the experimental site in 2013, 2014, and 2015. The sum of precipitation (in mm) is shown for individual days in (A) 2013, (B) 2014, and (C) 2015. Average daily air temperature (black line) and daily minimum and maximum temperature (gray shaded area) (in °C) is shown for (D) 2013, (E) 2014, and (F) 2015.

Weather data for all three seasons were available from nearby weather stations. The air temperature and precipitation data (Fig. 1) were obtained from Willard Airport (http://mrcc.isws.illinois.edu/CLIMATE/, last accessed 2 August 2016), 1 km west of the site, and the photosynthetically active radiation (PAR; μmol photons m^−2^ s^−1^) data from the University of Illinois Energy Farm (http://www.energybiosciencesinstitute.org/content/ebi-weather, last accessed 22 June 2016), 2 km to the east. Air temperature during the June–October growing season was close to the 20 year average (19.9 °C) in all three years. In comparison with the 20 year averages of the annual sum of precipitation (922 mm) and the sum of precipitation during the June–October growing season (446 mm), precipitation was slightly lower in 2013 and higher in 2014 and 2015 (Table 1). PAR was very similar in all three growing seasons, with maximum/mean values of 2190/737 μmol m^−2^ s^−1^ (2013), 2388/721 μmol m^−2^ s^−1^ (2014), and 2236/724 μmol m^−2^ s^−1^ (2015).

### Plant material

*Glycine max* (L.) Merr. cv. ‘Thorne’ was previously transformed (Bihmidine, 2012) with the cyanobacterial gene *FBP1* (*Synechococcus*, strain PCC7942) using the *Agrobacterium*-mediated method (Hinchee *et al.*, 1988; Clemente, 1997) to test the effect of *FBP1* on soybean leaf *A*. The *FBP1* gene encodes the FBPase/SBPase bifunctional enzyme, which has the same enzymatic function as the two individual enzymes present in higher plants. The *FBP1* cassette contains the *FBP1* gene under the control of the *Peanut chlorotic streak caulimovirus* (pcisvflt36) promoter, coupled with the tobacco etch translational enhancer element, and attached to the pea Rubisco small subunit transit peptide for transport of the enzyme across the plastid membrane (see Supplementary Fig. S1 at *JXB* online). The transgene cassette was subcloned into the binary vector pPTN200 which harbors a Pnos bar cassette for selection. Greenhouse and field phenotyping and molecular characterizations are described in Bihmidine (2012).

Due to spatial constraints of the experimental area and statistical considerations, we compared a single transgenic line with the WT. We used the same homozygous line (480-8) selected previously (Bihmidine, 2012; Hay, 2012), as it showed consistent increases of photosynthetic rates over WT plants. The present study was performed using T_7_, T_8_, and T_9_ populations derived from homozygous T_3_ and T_4_ populations of this line. T_5_ and T_6_ populations were multiplied in the field in 2011 and 2012 to provide enough seed for the start of the 3 year experiment. WT and FS plants were grown side by side, and only seeds harvested from the control treatment were used for planting in the following growing season. The presence of the transgene was reconfirmed in these subsequent generations using quantitative reverse transcription–PCR (data not shown) coupled with western blotting to test for the encoded protein.

### Protein extraction and western blotting

The level of bifunctional FBP/SBPase protein was assessed in fully expanded leaves that were sampled in the field at noon on the same days when *in situ* gas exchange measurements were collected. Additionally, the levels of transketolase were assessed as a loading control, and levels of native SBPase were assessed to check if expression of the bifunctional enzyme affected levels of the native enzyme. Three leaflets from three different plants in each treatment were cut and immediately plunged into liquid nitrogen. Leaflets were taken from the most recently fully expanded leaves at the top of the canopy.

Samples were stored at −80 °C before grinding in liquid nitrogen, and the resulting powder was subsequently stored at −80 °C until analysis. Subsamples of 100 μl of powder were mixed with 600 μl of protein extraction buffer [50 mM HEPES-NaOH (pH 8.2); 5 mM MgCl_2_; 1 mM EDTA; 1 mM EGTA; 10% glycerol; 0.1% Triton X-100; 2 mM benzamidine; 2 mM aminocaproic acid; 0.5 mM phenylmethylsulphonyl fluoride; 10 mM DTT] using a pre-cooled mortar and pestle at 4 °C. The resulting suspension was clarified at 10000 *g* for 2 min at 4 °C, and the supernatant was transferred to separate tubes for protein quantification and western blotting. Protein quantification was assessed by Bradford Assay (Bradford Reagent B6916, Sigma-Aldrich). A 200 μl aliquot of 313 mM Tris–HCl (pH 6.8), 10% SDS, 25% glycerol, 25% 2-mercaptoethanol was added to the protein samples for western blotting, boiled, and diluted to a uniform protein concentration. Samples were loaded on an equal protein basis (10 μg of soluble protein) on a 4% stacking gel, separated using 10% (w/v) SDS–PAGE, and transferred using a Mini Trans-Blot cell (Bio-Rad) to a nitrocellulose blotting membrane (Amersham Protran). The membrane was blocked in 6% (w/v) skim milk (made from Marvel Original dried skimmed milk powder) in phosphate-buffered saline (PBS) and incubated with a primary antibody (bifunctional FBPase/SBPase, dilution 1:500; native SBPase, dilution 1:2000; or transketolase (TK), dilution 1:5000) in 3% skim milk in PBS at 4 °C overnight. SBPase polyclonal antibodies were raised in rabbits against *Arabidopsis* SBPase, and TK antibodies were raised against tobacco plastid TK as described in Henkes *et al.* (2001). Bifunctional FBP/SBPase polyclonal antibodies were raised against DRPRHKELIQEIRNAG-[C]-amide (Cambridge Research Biochemicals, Cleveland, UK). The membrane was rinsed with 300 ml of PBST, then placed on a shaker table and washed for 60 min in PBST (fresh PBST replaced every 20 min). Goat anti-rabbit antibody conjugated to horseradish peroxidase (Invitrogen, Thermo Fisher Scientific, Catalog#: 31466) was used at a 1:2500 dilution in 3% skim milk in PBS with 0.05% Tween-20 (PBST) and the membrane incubated for 1.5 h at 4 °C. After incubation, the membrane was placed on a shaker table and washed for 30 min in PBST (fresh PBST replaced every 10 min). Proteins were detected using the enhanced chemiluminescence detection reagent (Pierce ECL Western Blotting Substrate, ThermoFisher).

### Above-ground biomass, seed yield, harvest index, and growth parameters

After full maturity [R8, 95% of pods have reached mature pod color (Ritchie *et al.*, 1993)] and dry-down was complete, plants were harvested by hand to determine yield and total biomass. Above-ground biomass (AGB; g m^−2^), seed yield (SY; g m^−2^), harvest index (HI=SY/AGB; unitless), 200 seed weight (200 SW, g), and growth parameters (number of plants, mean plant height, number of nodes and pods per plant) were determined in all years. AGB comprises stems and pods only, as leaves have senesced and fallen by the time of harvest. In all years, plants were cut from a 1 m length of row from the undisturbed middle two rows of each genotype subplot to determine the number of plants, mean plant height, number of nodes, and pods per plant, AGB, and HI. Pods and stems were separated by hand and dried at 65 °C to constant weight. After determination of stem and pod weight, the seeds were separated from the pods using a belt thresher and SY was determined to calculate HI. These seeds were also used to obtain individual seed weight (SW) by counting and weighing 200 seeds of each genotype in each subplot. In 2014 and 2015, we additionally harvested all plants along a total row length of 2.2 m to obtain a more robust estimate of SY, and this was added to SY from the 1 m row for statistical analysis. SY and AGB data were converted from g m^−1^ to g m^−2^.

### *In situ* gas exchange measurements

Gas exchange measurements were conducted on 2 (2013), 5 (2014), and 4 (2015) d during the growing seasons (Table 2), covering vegetative and reproductive stages from V3 to V5 (Third to Fifth-Node Stage) to R6 (Full Seed) based on the soybean development classifications of Ritchie *et al.* (1993). Measurements on DOY (day of year) 255 in 2014 were excluded from the analysis as leaves were starting to senesce. Leaf-level gas exchange systems (LI-6400XT; LI-COR Inc., Lincoln, NE, USA) coupled with the LI-6400-40 Leaf Chamber Fluorometer (LI-COR, Inc.) were used for these measurements. Measurements were conducted on plants in the two outer rows. The diurnal course of the photosynthetic carbon assimilation rate (*A*, μmol m^−2^ s^−1^) was determined on the middle leaflet of the youngest fully expanded trifoliate on 2–3 plants per genotype on each subplot at three time points between 9:00 h and 17:00 h. Conditions in the leaf chamber (block temperature, reference [CO_2_], PAR) were set at the beginning of each time point, based on ambient conditions. Block temperature was set according to air temperature reported from a nearby weather station and increased by the target temperature difference of +3.5 °C for the heated treatments. The reference [CO_2_] was set to 410 μmol mol^−1^ and 610 μmol mol^−1^ for the ambient and elevated [CO_2_] plots in order to match plot treatment conditions within the leaf chamber after the leaves lowered the [CO_2_]. Ambient PAR was measured immediately before each time point using the LI-190 installed on the LI-6400. The output of the chamber light-emitting diodes (LEDs) was set to deliver the same PAR and was maintained at these values throughout the course of all measurements at that time point. Relative humidity (RH) in the leaf chamber was not controlled directly, but was targeted to be kept between 50% and 70% during the measurement, which was achieved for 90% of the data. Environmental conditions during the *in situ* measurements are presented in Table 2.

In 2013 each of the four LI-6400 systems was randomly assigned to one block and the measurements conducted on the subplots in randomized order. In 2014 and 2015, two teams, each equipped with two LI-6400 gas exchange systems assigned at random, conducted the measurements in parallel per block, with one instrument assigned to the heated subplot and the other to the unheated subplot. At each time point, instruments stayed assigned to the same temperature treatment within a block but were switched on transition to the next block to avoid confounding any undetected instrument difference with a treatment. All measurements for a given time point were completed within 1.5–2 h.

### *A–* *C*_i_ curves: measurements and model fitting

The maximum carboxylation rate of Rubisco (*V*_c,max_) and *J*_max_ were determined from *A–C*_i_ curve measurements of *A* at varying intercellular [CO_2_] following the protocol of Bernacchi *et al.* (2005) on leaves that were collected in the field pre-dawn. *A*–*C*_i_ curve measurements were conducted within 2 d before or after the *in situ* gas exchange measurements. Measurements on DOY 255 in 2014 were excluded from the analysis as leaves were starting to senesce. The petiole of the youngest fully expanded leaf from two plants per subplot was cut close to the stem and then immediately re-cut in water. Leaves were transported to the lab in an opaque box and were kept at 20 °C and under low-light conditions (PAR <10 μmol photons m^−2^ s^−1^) until measurement. Leaves were exposed to high light (1000 μmol m^−2^ s^−1^ PAR) for 10–15 min before clamping the leaf cuvette onto the middle leaflet. Conditions in the leaf cuvette were set to reflect the treatment [CO_2_] (410 μmol mol^−1^ or 610 μmol mol^−1^), the light level was set to 1500 μmol m^−2^ s^−1^, leaf temperature was controlled at 25 °C (±0.3 °C SD), and mean vapor pressure deficit during the measurements was 1.4 kPa (±0.2 kPa SD). After ~5 min, the leaf had reached steady-state *A* and the *A*–*C*_i_ curve autoprogram was initiated. [CO_2_] was decreased stepwise to 50 μmol mol^−1^ or 100 μmol mol^−1^, then increased back to the starting value (410 μmol mol^−1^ or 610 μmol mol^−1^) and then stepwise up to 1100 μmol mol^−1^ or 1500 μmol mol^−1^, depending on the CO_2_ treatment and developmental stage. Measurements of *A* were recorded at each [CO_2_] set point after stability was reached. *V*_c,max@25 °C_ and *J*_max@25 °C_ were derived from a curve-fitting procedure of the underlying biochemical models (Farquhar *et al.*, 1980; von Caemmerer, 2000), following the procedures described by Long and Bernacchi (2003).

### Statistical analysis

Gas exchange and yield data were analyzed using a mixed-model analysis of variance (PROC MIXED, SAS 9.4), taking into account the split-plot design of the experiment. The analyses were conducted separately for individual years. The model for the diurnal gas exchange data was analyzed separately by time of day (morning, midday, afternoon) and included the fixed factors [CO_2_] (ambient, elevated), temperature (control, heated), genotype (WT, FS), and day of the measurement (day of year, DOY), which was included as a repeated measure. Block was included as a random factor. The model for end of season yield data included the fixed factors [CO_2_], temperature, and genotype, and block as random factor. Significant differences between least square means for *a priori* determined comparisons were analyzed using post-hoc tests (LSMEANS, SAS 9.4). Probability for statistical significance was set at *P*<0.1 *a priori* to reduce the possibility of type II errors.

## Results

### Characterization of WT and FBP/SBPase-expressing plants by western blotting

**Fig. 2.**
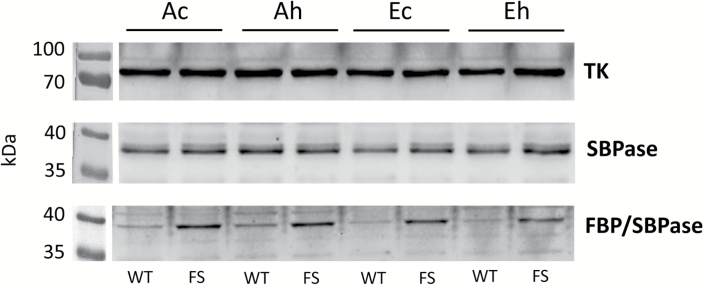
Western blot analysis of leaf protein extracts from wild-type (WT) and bifunctional FBP/SBPase-expressing (FS) plants. Proteins were extracted from 2014 leaf tissue samples (R5, beginning seed developmental stage) of plants grown under the ‘*Ac*’ (400 μmol mol^−1^ [CO_2_], control temperature), ‘*Ah*’ (400 μmol mol^−1^ [CO_2_], heated +3.5 °C), ‘*Ec*’ (600 μmol mol^−1^ [CO_2_], control temperature), and ‘*Eh*’ (600 μmol mol^−1^ [CO_2_], heated +3.5 °C) treatments. A total of three leaflets (from three different plants) were combined per sample. The blot was probed at the same time with polyclonal antibodies raised against transketolase (TK) and native SBPase, and was reprobed using a polyclonal antibody raised against bifunctional FBP/SBPase after the blot was stripped. Gels were loaded on an equal protein basis. Blot marker images were cropped and pasted onto the corresponding chemiluminescence images using the editing function of the FusionCapt Advance software.

Western blotting showed that the bifunctional FBP/SBPase was present in the transgenic plants and appeared to have little effect on native SPBase levels (Fig. 2). Bands matched the expected sizes of the polypeptides of ~72 kDa for TK, ~36 kDa for SBPase, and ~38 kDa for bifunctional FBP/SBPase. The FBP/SBPase antibody showed cross-reactivity and non-specific binding, but the strongest bands were clearly only observed for the expected size of the bifunctional FBP/SBPase protein in the transgenic plants. Levels of expression did not appear to vary based on treatment (Fig. 2).

### FS plants had higher photosynthesis than WT plants, especially under high light and high temperature conditions

**Fig. 3.**
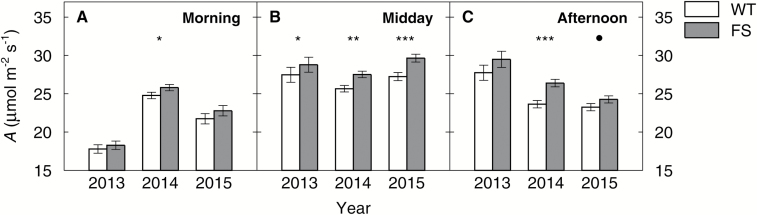
Average photosynthetic rates (*A*, μmol m^−2^ s^−1^) of the wild-type (WT) and bifunctional FBP/SBPase-expressing (FS) plants in comparison. Morning (A), midday (B), and afternoon (C) *in situ* gas exchange measurements were pooled for all four treatments (ambient/elevated CO_2_, control/heated plots) and for all sampling days in the respective years. Temperature, reference [CO_2_], and light (photosynthetic active radiation, PAR) in the LI-6400XT leaf chamber were set to match ambient conditions at the beginning of the measurements and the values were maintained throughout the course of all measurements at the respective time point. Relative humidity (RH) was kept between 50% and 70% during the measurements. Environmental conditions during the *in situ* measurements are presented in Table 2. Error bars are ±SE of the LS means estimate as derived from a repeated measures ANOVA (Supplementary Table S1). Symbols mark significant differences between WT (empty bars) and FS (gray bars) (• <0.1, * <0.05, ** <0.01, *** <0.001). Note that the *y*-axis does not start at zero.

*In situ* gas exchange measurements showed no significant difference in *A* between genotypes during the morning (Fig. 3A), with the exception of 2014 where FS plants had 11% and 14% higher *A* than the WT in the *Ac* and the *Ec* treatment on DOY 238 (Supplementary Fig. S2). During midday measurements, FS plants always had a higher *A* than the WT by 5% (2013), 7% (2014), and 9% (2015) (Fig. 3B). There was a significant DOY×genotype interaction for midday *A* in all years (Supplementary Table S1). FS plants tended to have higher midday *A* than WT plants during the middle of the seasons, but no significant differences were observed early and late in the seasons (Supplementary Fig. S2). During afternoon measurements, FS plants had a 12% (2014) and 4% (2015) higher *A* than the WT, but no significant differences were observed for 2013 (Fig. 3C). For a summary of the ANOVA statistics, see Supplementary Table S1.

Environmental conditions on the days of *in situ* gas exchange measurements varied greatly, with PAR ranging from as low as 250 μmol m^−2^ s^−1^ up to 2000 μmol m^−2^ s^−1^ and air temperature from 13 °C to 32 °C (Table 2). *A* of FS plants was significantly higher than *A* of WT plants on certain days, but the response was not observed for all time points and treatments. Light levels and air temperature tended to be higher at the times when significant differences between FS and WT plants (Δ*A*=*A*_FS_−*A*_WT_) were observed (Table 2). Multiple regression analysis (Δ*A*=T_air_+PAR) with the pooled data for all seasons and developmental stages revealed slight increases of Δ*A* with PAR (1.4 μmol CO_2_ m^−2^ s^−1^/1000 μmol photons m^−2^ s^−1^, *P*<0.01) and T_air_ (0.8 μmol CO_2_ m^−2^ s^−1^/10 °C, *P*<0.1), but overall variance was high (*R*^2^=0.1, *P*<0.001) (Supplementary Fig. S3).

**Table 2.** Day of year (DOY) and time points (9:00 h, 12:00 h, and 15:00 h) on which *in situ* gas exchange measurements were conducted in the three growing seasons and the corresponding developmental stages and environmental conditions (air temperature, T_air_; photosynthetic active radiation, PAR)

DOY	Growth stage	T_air_ (°C) control/heated			PAR (μmol photons m^−2^ s^−1^)		
		9:00	12:00	15:00	9:00	12:00	15:00
2013							
192	~V3	22.0/25.5	25.0/28.5	25.0/28.5	1100	2000	1400
**241**	**R5/R6**	22.0/25.5	**26.0**/**29.5**	28.5/32.0	250	**900**	1700
2014							
**199**	**V4/V5**	20.0/23.5	27.0/30.5	**24.0**/**27.5**	1000	500	**1550**
218	R1/R2	22.0/25.5	24.5/28.0	25.5/29.0	600	500	400
**227**	**R3**	18.0/21.5	**20.5**/**24.0**	24.5/28.0	1000	**1500**	850
**238**	**R5**	**25.5**/**29.0**	**30.0**/**33.5**	**32.0**/**35.5**	**1150**	**1800**	**1000**
255	R6	13.0/16.5	15.0/18.5	15.0/18.5	400	600	500
2015							
184	V3/V4	19.0/22.5	23.0/26.5	22.0/25.5	1000	930	390
196	R1	18.0/21.5	23.0/26.5	26.0/29.5	600	2000	1500
**216**	**R4**	21.0/24.5	**26.0**/**29.5**	27.0/30.5	1000	**1800**	1200
237	R6	16.0/19.5	21.0/24.5	22.0/25.5	1200	1800	1650
Mean ±SE	ns	19.1 ± 0.9	22.6 ± 1.5	23.9 ± 1.3	815 ± 103	500 ± 212	1066 ± 180
	sign.	25.5	25.6 ± 2	28 ± 4	1150	1190 ± 270	1275 ± 275

Bold font indicates that significant differences (*P*<0.1) in photosynthetic rate (*A*) between genotypes were observed at these points in time.

Mean values for T_air_ and PAR where no significant (ns) or significant (sign.) differences were observed are given at the bottom of the table. When differences were significant, *A* was always higher for the FS plants.

V3, V4, V5, third, fourth, and fifth node stage; R1, beginning bloom; R2, full bloom; R3, beginning pod, R4, full pod; R5, beginning seed; R6, full seed (Ritchie *et al.*, 1993)

### FS plants had higher *V*_c,max_ and *J*_max_, especially during late reproductive stages

**Fig. 4.**
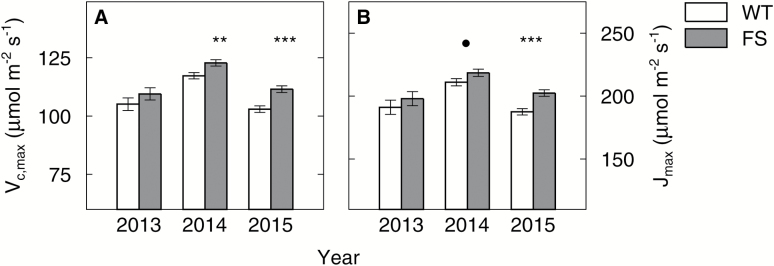
Mean *V*_c,max_ and *J*_max_ of the wild-type (WT) and bifunctional FBP/SBPase-expressing (FS) plants in comparison. *V*_c,max_ (A) and *J*_max_ (B) were derived from *A*–*C*_i_ curves conducted at a leaf temperature of 25 °C and the data were pooled for all four treatments (ambient/elevated CO_2_, control/heated plots) and for all sampling days in the respective years. Error bars are ±SE of the LS means estimate as derived from a repeated measures ANOVA. Symbols mark significant differences between the WT (empty bars) and FS (gray bars) (• <0.1, ** <0.01 *** <0.001). Note that the *y*-axis does not start at zero.

There was a significant main effect of genotype on *V*_c,max_ (Fig. 4A) and *J*_max_ (Fig. 4B) with significantly higher values for FS plants than WT plants in 2014 (*V*_c,max_+5.5 μmol m^−2^ s^−1^, *P*<0.01; *J*_max_+7.5 μmol m^−2^ s^−1^, *P*<0.1) and 2015 (*V*_c,max_+8.6 μmol m^−2^ s^−1^, *P*<0.001; *J*_max_+14.9 μmol m^−2^ s^−1^, *P*<0.001). The differences in 2013 were not significant, but the trend toward higher values for the FS plants was still present. Higher values of *V*_c,max_ and *J*_max_ of FS plants in comparison with the WT were particularly observed during the second half of the growing seasons. The biggest differences occurred at the R5/R6 developmental stage, but were not consistently observed under all treatments (Supplementry Fig. S4).

### FS plants maintained seed yield under the elevated [CO_2_] and heat treatment while WT plants had significantly reduced seed yield

**Fig. 5.**
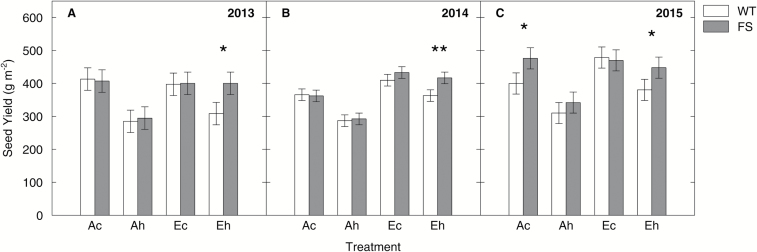
Total seed yield (g m^−2^) of the wild-type (WT) and bifunctional FBP/SBPase-expressing (FS) plants in comparison in the three growing seasons. Plants were grown under the ‘*Ac*’ (400 μmol mol^−1^ [CO_2_], control temperature), ‘*Ah*’ (400 μmol mol^−1^ [CO_2_], heated +3.5 °C), ‘*Ec*’ (600 μmol mol^−1^ [CO_2_], control temperature), and ‘*Eh*’ (600 μmol mol^−1^ [CO_2_], heated +3.5 °C) treatments at the Soy-T-FACE experiment. Plants were harvested along a total row length of 1 m in 2013 (A) and of 3.2 m in 2014 (B) and 2015 (C), and seed yield was converted into g m^−2^. Error bars are ±SE of the LS means estimate as derived from the complete block ANOVA (Table 3) Symbols mark significant differences between the WT (empty bars) and FS (gray bars) (* <0.05, ** <0.01).

There was a significant main effect of genotype on SY in 2014 and 2015 (Table 3), and pairwise comparisons of genotypes within treatments showed a significant difference under the *Eh* treatment in all years (Fig. 5). This was related to significant reductions between 11% and 22% of WT SY under the *Eh* treatment compared with the *Ec* treatment, while SY of FS plants was unchanged. In 2015 (Fig. 5C), FS plants also had higher yield under the *Ac* treatment in comparison with the WT. Also in 2015, there was a significant interaction of genotype with [CO_2_] and temperature on SY (Table 3), consistent with the hypothesis that the FS plants would have a particular advantage under conditions of combined elevation of temperature and [CO_2_]. Both WT and FS plants had generally higher SY under the elevated [CO_2_] treatments in comparison with ambient [CO_2_] in 2014 and 2015 (*P*<0.05) but not in 2013 (Table 3; Fig. 5A). In all three growing seasons, elevated temperature significantly decreased SY by between 11% and 31%, depending on year and [CO_2_], with the exception of the FS plants in the *Eh* treatment, where no significant decline was observed (Fig. 5).

**Table 3.** Seasonal complete block ANOVA of total seed yield (SY), weight of 200 seeds (SW), above-ground biomass (AGB), stem and branches (ST+BN), harvest index (HI), number of pods per plant (POD), stem height (STH), number of nodes per plant (NODE) for the main effects [CO_2_] (400 μmol mol^−1^, 600 μmol mol^−1^), temperature ‘Temp.’ (control, heat), and genotype (WT, FS) and their interaction terms

Parameter, unit		[CO_2_]	Temp.	Genotype	[CO_2_]× Temp.	[CO_2_]× Genotype	Temp.× Genotype	[CO_2_]× Temp.× Geno type
df		3	6	12	6	12	12	12
2013								
SY	g m^−2^	ns	**0.032**	ns	ns	ns	ns	ns
SW	g	ns	**0.001**	ns	**0.024**	ns	ns	ns
AGB	g m^−2^	**0.099**	**0.070**	ns	ns	ns	ns	ns
ST+BN	g m^−2^	**0.009**	**0.042**	**0.020**	ns	ns	ns	ns
HI		**0.066**	**0.005**	**0.008**	**0.078**	ns	ns	ns
POD	No. per plant	ns	ns	**0.015**	ns	ns	ns	ns
STH	m	**0.014**	**0.003**	**0.047**	ns	**0.031**	ns	ns
NODE	No. per plant	ns	ns	ns	ns	**0.021**	ns	ns
2014								
SY	g m^−2^	**0.014**	**0.001**	**0.011**	**0.065**	**0.015**	ns	ns
SW	g	ns	ns	**0.085**	ns	ns	ns	ns
AGB	g m^−2^	**0.034**	**0.026**	**0.058**	**0.004**	ns	ns	ns
ST+BN	g m^−2^	**0.029**	**0.030**	ns	**0.080**	ns	ns	ns
HI		ns	**0.097**	ns	ns	ns	ns	ns
POD	No. per plant	**0.053**	**0.066**	ns	ns	ns	ns	ns
STH	m	**0.082**	**0.001**	**0.024**	ns	ns	**0.085**	ns
NODE	No. per plant	ns	**0.002**	**0.028**	ns	ns	ns	ns
2015								
SY	g m^−2^	**0.024**	**0.001**	**0.014**	ns	ns	ns	**0.061**
SW	g	ns	ns	ns	ns	ns	ns	ns
AGB	g m^−2^	**0.008**	**0.001**	ns	**0.017**	ns	ns	**0.002**
ST+BN	g m^−2^	**0.008**	**0.036**	**<0.0001**	**0.036**	ns	**0.018**	**0.006**
HI		ns	**0.002**	**0.001**	ns	ns	**0.049**	ns
POD	No. per plant	**0.009**	**0.006**	**0.007**	ns	**0.037**	ns	**0.043**
STH	m	**0.004**	ns	**<0.0001**	ns	ns	ns	ns
NODE	No. per plant	**0.013**	ns	**0.053**	ns	ns	**0.094**	ns

Only interaction terms with significant effects are listed in the table.

Values in the table are *P*-values; significance was set as *P*<0.1.

df, degrees of freedom; ns, not significant.

### Genotype did not have consistent effects on 200 seed weight, above-ground biomass, or harvest index

**Fig. 6.**
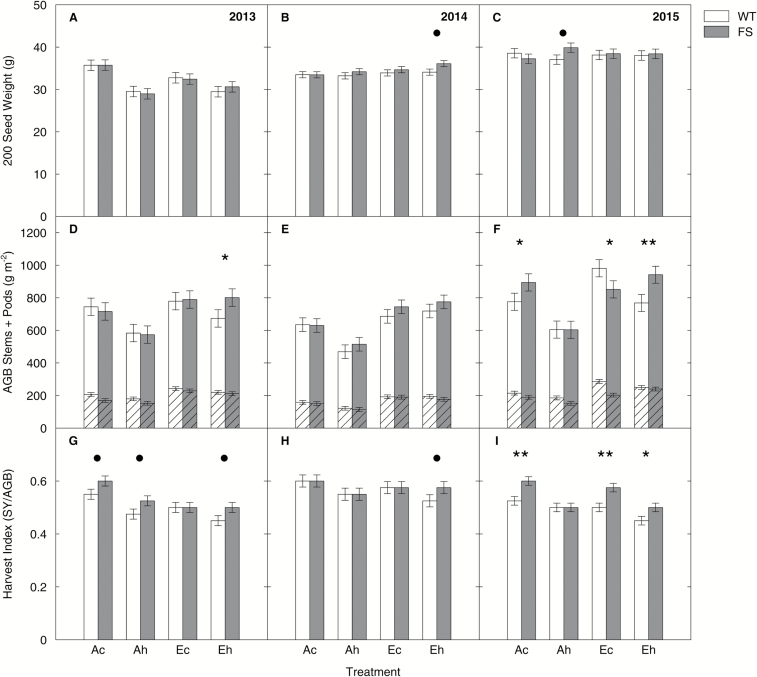
Seed weight, above-ground biomass, and harvest index of the wild-type (WT) and bifunctional FBP/SBPase-expressing (FS) plants in comparison in the three growing seasons. Seed weight of 200 seeds (in g), above-ground biomass (AGB, in g m^−2^) comprised of stems and branches (dashed area) and pods (undashed area), and harvest index [HI; seed yield (SY)/AGB] were derived from plants grown under the ‘*Ac*’ (400 μmol mol^−1^ [CO_2_], control temperature), ‘*Ah*’ (400 μmol mol^−1^ [CO_2_], heated +3.5 °C), ‘*Ec*’ (600 μmol mol^−1^ [CO_2_], control temperature), and ‘*Eh*’ (600 μmol mol^−1^ [CO_2_], heated to +3.5 °C) treatments at the Soy-T-FACE experiment. Data are from a representative sampling along 1 m length of a middle row within each subplot in the three growing seasons. Error bars are ±SE of the LS means estimate as derived from the complete block ANOVA (Table 3). Symbols mark significant differences between the WT (empty bars) and FS (gray bars) (• <0.1, * <0.05, ** <0.01).

There were no differences in 200 seed weight (SW) between genotypes with the exception of a slightly bigger SW of FS plants in comparison with the WT (*P*<0.1) in the *Eh* treatment in 2014 (Fig. 6B) and the *Ah* treatment in 2015 (Fig. 6C). Significant differences in pairwise comparisons for AGB between genotypes within treatments were found for the *Eh* treatment in 2013 (FS+19%, *P*<0.05, Fig. 6D) and the *Ac* (FS+15%, *P*<0.05), *Ec* (FS−13%, *P*<0.05), and *Eh* (FS+23%, *P*<0.01) treatment in 2015 (Fig. 6F). The high AGB of the WT under the *Ec* treatment in 2015 was related to a very high stem weight in 2015 (Fig. 6F). Over all treatments, WT plants showed a lower HI than FS plants in 2013 (−7%, *P*<0.01) and 2015 (−9%, *P*<0.001) but not in 2014 (Table 3; Fig. 6G-I).

### FS plants were shorter and tended to have more pods per plant than the WT, but did not differ consistently in the number of nodes per plant at the time of harvest

**Fig. 7.**
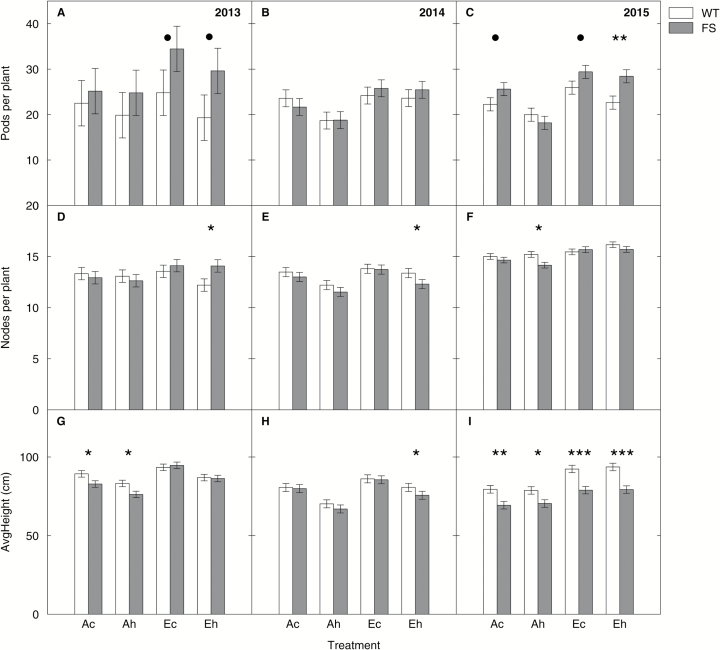
Pods per plant, nodes per plant, and average height of the wild-type (WT) and bifunctional FBP/SBPase-expressing (FS) plants in comparison in the three growing seasons. Data were derived from plants grown under the ‘*Ac*’ (400 μmol mol^−1^ [CO_2_], control temperature), ‘*Ah*’ (400 μmol mol^−1^ [CO_2_], heated +3.5 °C), ‘*Ec*’ (600 μmol mol^−1^ [CO_2_], control temperature), and ‘*Eh*’ (600 μmol mol^−1^ [CO_2_], heated +3.5 °C) treatments at the Soy-T-FACE experiment. Data are from a representative sampling along 1 m length of a middle row within each subplot in the three growing seasons. Error bars are ±SE of the LS means estimate as derived from the complete block ANOVA (Table 3). Symbols mark significant differences between the WT (empty bars) and FS (gray bars) (• <0.1, * <0.05. ** <0.01 *** <0.001).

A significant main effect of genotype (Table 3) on the number of pods per plant was observed in 2013 (Fig. 7A) and 2015 (Fig. 7C), with on average 7 (2013, *P*<0.05) and 3 (2015, *P*<0.01) more pods on the FS plants. There was a significant main effect of genotype on number of nodes in 2014 (Fig. 7E, *P*<0.05) and 2015 (Fig. 7F, *P*<0.1) but pairwise comparisons between genotypes showed no clear treatment effect. A significant main effect of genotype on plant height (Fig. 7G–I) was observed in all three years, with FS plants being on average smaller than WT plants (2013, −3.2 cm, *P*<0.05; 2014, −2.4 cm, *P*<0.05; 2015, −11.6 cm, *P*<0.001).

## Discussion

The objective of this research was to determine the potential for soybean overexpressing SBPase to be better adapted to growth in a future high CO_2_ atmosphere and warmer climate as compared with current conditions. This study builds upon previous research showing that SBPase overexpression leads to higher *A* and higher biomass production in tobacco (Miyagawa *et al.*, 2001; Lefebvre *et al.*, 2005; Rosenthal *et al.*, 2011; Simkin *et al.*, 2015), which we now extend to show an effect on yield in a major food crop and under simulated climate change conditions in the field. We hypothesized that expressing a cyanobacterial, bifunctional FBP/SBPase in soybean would lead to higher *A* and higher yield, particularly under conditions that favor RuBP regeneration limitation, namely future elevation of [CO_2_] and temperature. This hypothesis is supported by the consistent observation across all three years that the transgenic plants in the *Eh* treatment were able to maintain SY at the *Ec* treatment levels, while WT plants showed significant losses in SY (Fig. 5). This suggests that expression of FBP/SBPase in soybean may help to prevent yield losses in the likely scenario where [CO_2_] and temperatures will increase together under future climate conditions.

Studies with tobacco plants showed that either overexpression of SBPase or expression of the bifunctional FBP/SBPase enzyme generally enhanced *A* of the transgenic plants in comparison with the WT under both ambient (Miyagawa *et al.*, 2001; Lefebvre *et al.*, 2005) and elevated (Rosenthal *et al.*, 2011) [CO_2_]. Similarly, we observed that FS plants tended to have higher photosynthetic rates, but the significant genotype×DOY interactions suggested an effect of environmental conditions and/or developmental stage on the difference between FS and WT *A*. Our *in situ* gas exchange measurements throughout the three seasons covered a wide range of light levels and temperatures, with light levels ranging from 250 μmol photons m^−2^ s^−1^ to 2000 μmol photons m^−2^ s^−1^ and temperatures ranging from 13 °C to 35.5 °C. Because SBPase is only potentially limiting when *A* is RuBP limited, a positive effect of SBPase on *A* was only expected under conditions consistent with RuBP regeneration limitation. Higher *A* of FS plants in comparison with the WT predominantly occurred during peak light and higher temperatures, and this observation is consistent with other studies. SBPase-overexpressing tobacco plants had up to 12% higher midday *A* when grown in a controlled-environment greenhouse under high light (Lefebvre *et al.*, 2005) and up to 14% higher midday *A* when grown in the field (Rosenthal *et al.*, 2011) in comparison with WT tobacco plants. However, tobacco plants overexpressing SBPase grown under low light conditions and short-day conditions in the greenhouse in the winter did not show increases in growth and *A* relative to WT tobacco plants (Lefebvre *et al.*, 2005).

In addition to the expected increase in *J*_max_, *V*_c,max_ was also increased in the FS plants (Fig. 4), indicating an increase in SBPase activity having a pleiotropic effect on the activity of Rubisco *in vivo.* Although the increase in *V*_c,max_ was lower than the increase in *J*_max_, it would result in some increase in *A* even at low [CO_2_]. This may explain why higher *A* was not only observed under the *Eh* treatment as hypothesized (Supplementary Fig. S2). This effect on *V*_c,max_ was also observed by Lefebvre *et al.* (2005) for tobacco overexpressing SBPase grown in a controlled-environment greenhouse (light levels of 600–1600 μmol photons m^−2^ s^−1^), but Rosenthal *et al.* (2011) did not find any differences in *V*_c,max_ between field-grown WT and SBPase-overexpressing tobacco plants. The Rubisco activation state may be influenced by a mechanism described by Rokka *et al.* (2001) and observed by Feng *et al*. (2007a, b) in rice plants overexpressing SBPase, which maintained *A* and growth rate under heat and osmotic stress and recovered faster relative to WT plants. Feng *et al.* (2007a, b) suggest that overexpression of SBPase prevented the sequestration of Rubisco activase (RCA) to the thylakoid membranes, thus keeping the Rubisco activation state high. This may help explain the observed consistent yield of the FS plants under the high temperature and elevated [CO_2_]treatment, but it is unclear why this proposed mechanism, if it occurred, was not beneficial in the heat treatment under ambient [CO_2_].

The measured rates of *A* in our experiment did not always differ under high light and high temperature conditions, indicating that factors beyond temperature and light may play a role. Rosenthal *et al.* (2011) only observed significant differences in *A* during the vegetative stage and not during the reproductive stage in the field. Under controlled environment conditions, Lefebvre *et al.* (2005) also found an effect primarily during early growth for tobacco overexpressing SBPase, while the tobacco plants expressing FBP/SBPase exhibited higher differences in growth rates in older plants (Miyagawa *et al.*, 2001). The field-grown, FBP/SBPase-expressing soybean plants in our study exhibited greater differences in *A* with maturation. We observed no differences in *A* during the early vegetative developmental stages (V3–V5) even under high light levels of up to 2000 μmol m^−2^ s^−1^. Differences here compared with previous work might result from the use of different species (tobacco versus soybean), the type of promoter used [*Cauliflower mosaic virus* (CaMV) 35S versus *Peanut chlorotic streak caulimovirus* (PCISV)], differences between the SBPase and FBP/SBPase enzyme, and/or the growth conditions (field versus controlled environment).

Higher *A* in the tobacco plants (over)expressing either SBPase or the bifunctional FBP/SBPase enzyme translated into higher biomass in all three previous studies (Miyagawa *et al.*, 2001; Lefebvre *et al.*, 2005; Rosenthal *et al.*, 2011). Here, AGB of the FS soybeans did not show consistent increases over the WT, but as leaf biomass was not included in this measure, our results cannot be compared directly with those from tobacco. Lefebvre *et al.* (2005) also reported a significant increase in stem height of the SBPase-overexpressing tobacco plants, while stem height of our FS soybean plants did not differ (2013 and 2015) from the WT or was even significantly smaller (2015). However, for grain crops such as soybean, increases in SY instead of total AGB are of agronomic interest. Assuming a constant carbon (C) concentration, a higher SY requires either more C to be fixed or that more of the fixed C is partitioned into seeds. In the first case, a correlation between the rate of *A* and SY would be expected. Modeling (Zhu *et al.*, 2007) and previous empirical studies (Harrison *et al.*, 1998; Miyagawa *et al.*, 2001; Lefebvre *et al.*, 2005; Rosenthal *et al.*, 2011) suggest that overexpression of SBPase increases C flow through the PCR cycle, thus in theory making more C available for seed production. However, despite our observation of higher *A* of FS soybean plants throughout all seasons particularly under high light and high temperature conditions (Table 2, Supplementary Fig. S2), significant differences in yield between FS and WT plants were only observed on the *Eh* treatment.

These differences in the responses of *A* and yield of the transgenic plants in comparison with the WT between years and within seasons highlight the complexity of relating these traits under variable environmental conditions. Increases in *A* do not necessarily translate to higher biomass or yield (Long *et al.*, 2006) and increased allocation of carbon may have occurred to plant organs that were not measured in this experiment (roots, root nodules, leaves) or may have been offset by enhanced respiration. Under the combined heat and elevated [CO_2_] treatment (*Eh*), FS plants maintained SY at the same level as under the *Ec* (control temperature and elevated [CO_2_]) treatment, while WT SY was reduced by 11% to 22% in the three years. This difference was related to a higher number of pods of FS plants in 2013 and 2015 and a higher seed weight in 2014, indicating differences in allocation between the WT and FS plants. Accordingly, HI was also increased significantly for the FS plants. The observed consistent reduction of WT SY under heat treatment in our three season long experiment was similar to the effect reported by Ruiz-Vera *et al.* (2013) for another soybean cultivar (‘Pioneer 93B15’) in the 2011 season, suggesting that the yield loss under heat stress may not only be restricted to cultivar ‘Thorne’, which was used in our study.

In conclusion, we show that expression of the cyanobacterial, bifunctional FBP/SBPase generally leads to higher *A* in field-grown soybean and prevents yield losses under high [CO_2_] and high temperature, conditions that are expected for the near future. These findings are the first to show with a major food crop under open-air field conditions that manipulation of the PCR cycle can be used to mitigate the effects of global increases in temperature on yield under future elevated CO_2_ conditions.

## Supplementary Material

Supplementary Data

## Abbreviations:

A: net rate of CO_2_ uptake per unit leaf area (μmol m^−2^ s^−1^)
Ac: ambient CO_2_, control temperature treatment
[CO_2_]: CO_2_ concentration
Ah: ambient CO_2_, heated treatment
AGB: above-ground biomass
Ec: elevated CO_2_, control temperature treatment
Eh: elevated CO_2_, heated treatment
FBP/SBPase: fructose-1,6/sedoheptulose-1,7-bisphosphatase
FS plants: bifunctional FBP/SBPase-expressing plants
HI: harvest index
*J*_max_: RuBP regeneration capacity (μmol m^−2^ s^−1^)
PAR: photosynthetic active radiation (μmol m^−2^ s^−1^)
PCR: photosynthetic carbon reduction
RuBP: ribulose-1,5-bisphosphate
SBPase: sedoheptulose-1,7-bisphosphatase
SW: seed weight
SY: seed yield
*V*_c,max_: maximum carboxylation rate of Rubisco (μmol m^−2^ s^−1^)
WT: wild type.
