# Supplementary material for: Expression of cyanobacterial FBP/SBPase in soybean prevents yield depression under future climate conditions
Source: J Exp Bot. 2016 Dec 12;68(3):715–26. doi: 10.1093/jxb/erw435 (PMC5441901; doi:10.1093/jxb/erw435)
Supplement: Supplementary Data [file erw435_Supplementary_Data.zip › supplementary_figures_S1_S4_table_S1.pdf]

Supplementary Information

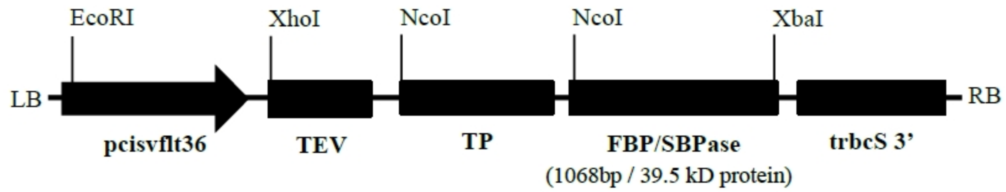

Supplementary Figure S1: *FBP1* gene construct used in the agrobacterium-mediated transformation of *Glycine max* cv. Thorne. The *FBP1* gene of interest is under the control of the peanut chlorotic streak caulimovirus full length transcript promoter (pcisvflt36), coupled with the tobacco etch virus translational enhancer (TEV) and the pea Rubisco small-subunit transit peptide (TP) and the terminator from the Rubisco small subunit (trbcS). LB, T-DNA left border; RB, T-DNA right border (from Bihmidine, 2012).

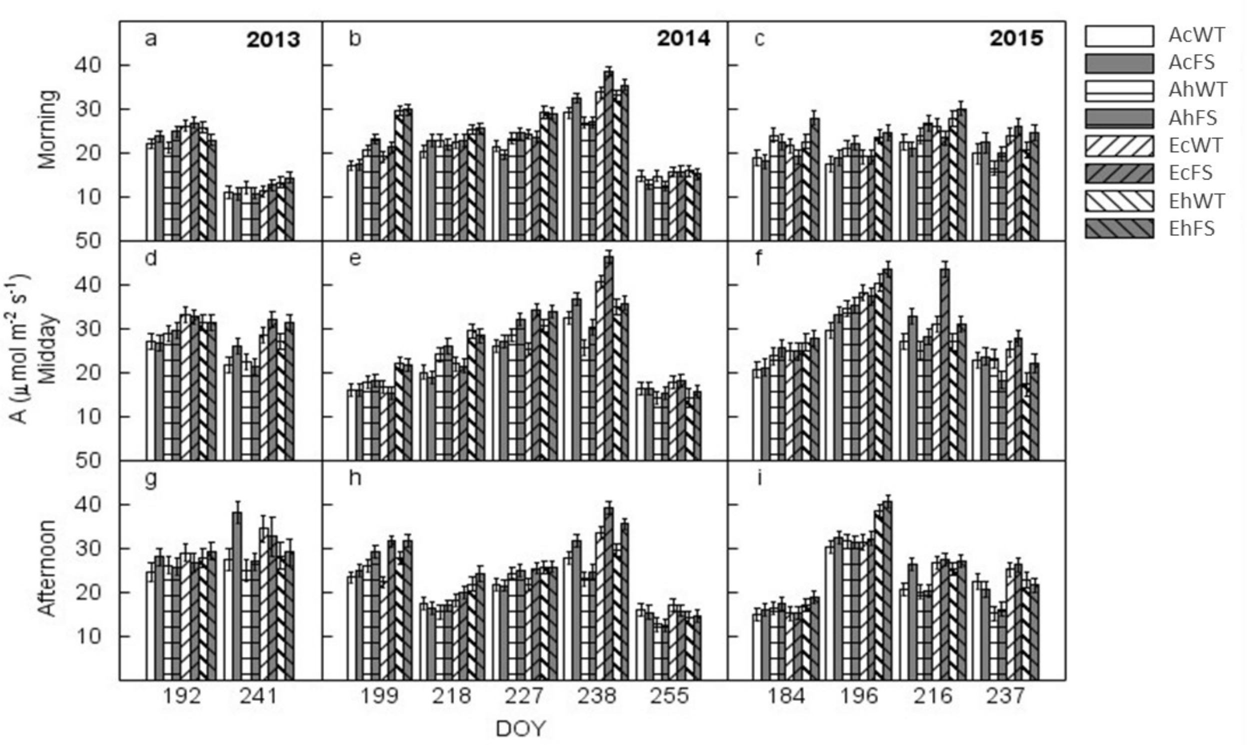

Supplementary Figure S2: Diurnal *in situ* photosynthesis (*A*) for all treatments and the two genotypes during the morning (a-c), midday (d-f) and afternoon measurements (g-h) in the three seasons 2013, 2014 and 2015. White bars represent results for WT and gray bars for FS plants, hatching represents the treatments (A = 400 μmol mol^{-1} CO_2, E = 600 μmol mol^{-1} CO_2, c = control temperature, h = heated). Error bars are ±SE of the estimate.

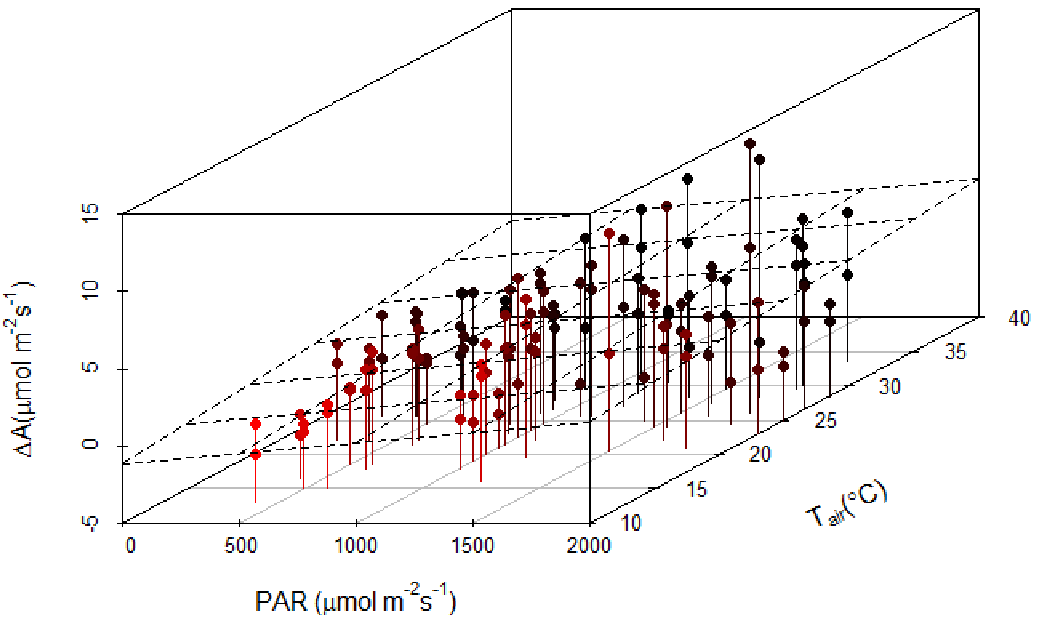

Supplementary Figure S3: 3D-Scatterplot of the mean difference between FS and WT photosynthesis (ΔA, μmol m^{-2} s^{-1}) as related to photosynthetic active radiation (PAR, μmol m^{-2} s^{-1}) and air temperature (T_{air}, °C). Points are drawn with a color gradient related to the *y*-coordinate (T_{air}), with red points having the lowest and black points the highest values. The plane represents the fitted values of the $\Delta A = T_{air} +$ PAR linear regression model.

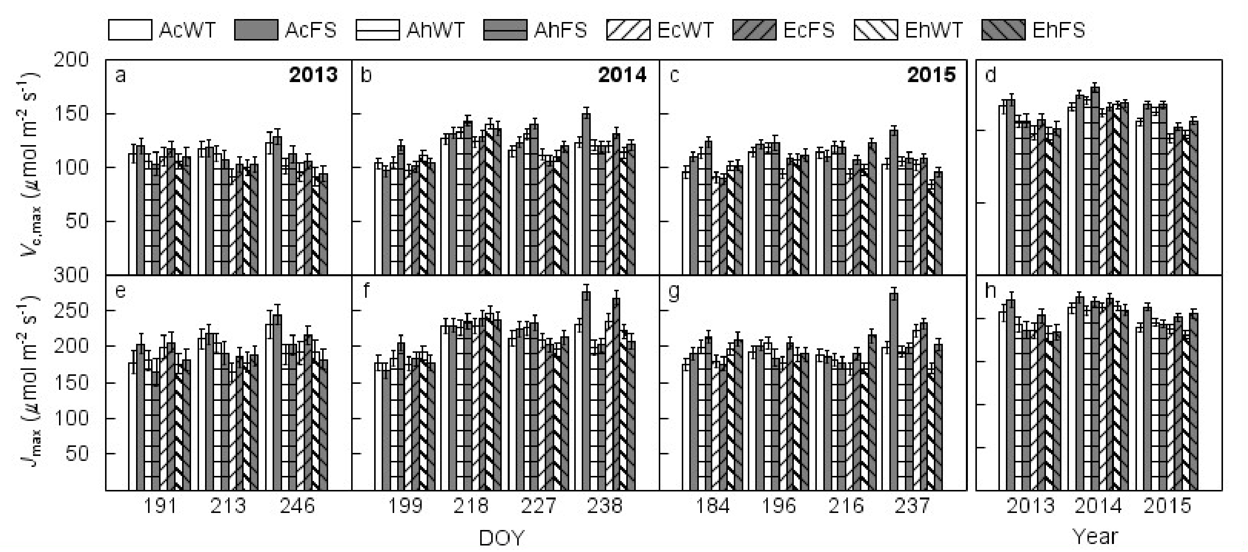

Supplementary Figure S4: $V_{c.max}$ and J_{max} separately for all treatments and sampling days (a-c, e-g) in the three years and averaged per year (d, h) for the WT (empty bars) and FS (gray bars) plants. White bars represent results for WT and gray bars for FS plants, hatching represents the treatments (A = 400 μmol mol^{-1} CO_2, E = 600 μmol mol^{-1} CO_2, c = control temperature, h = heated). Error bars are ±SE of the estimate, measurements were conducted at a leaf temperature of 25°C.

Supplementary Table S1: Results of the repeated measures ANOVA of the effects of CO_2, temperature, genotype and day of measurement (DOY) on the variation of morning, midday and afternoon photosynthesis (*A*). Data was analyzed separately by time of day (morning, midday, afternoon) and included the fixed factors [CO_2] (ambient, elevated), temperature (control, heated), genotype (WT, FS) and day of the measurement (day of year, DOY), which was included as a repeated measure. Block was included as a random factor. Only interaction terms with significant effects are listed in the table. Values in table are *p*-values, significance was set as $p < 0.1$, ns (not significant).

	DOY	[CO_2]	DOY*[CO_2]	Temp	DOY*Temp	[CO_2]*Temp	Geno-type	DOY*Geno-type	[CO_2]*Geno-type	DOY*[CO_2]*Geno-type	Temp*Geno-type	[CO_2]*Temp*Geno-type
2013												
Morning	**<0.0001**	**0.0117**	ns	ns	**0.09**	ns	ns	ns	ns	**0.0345**	ns	ns
Midday	**<0.0001**	**0.0379**	**0.0694**	ns	ns	ns	**0.0348**	**0.0829**	ns	ns	ns	ns
Aftern.	**0.0258**	ns	ns	**0.0758**	**0.0251**	ns	ns	ns	**0.0615**	ns	ns	**0.0432**
2014												
Morning	**<0.0001**	**0.005**	**0.0017**	**0.0068**	**<0.0001**	**0.0691**	ns	**0.0267**	ns	ns	ns	ns
Midday	**<0.0001**	**0.0011**	**0.0002**	ns	**<0.0001**	ns	**0.0008**	**0.0026**	ns	ns	ns	**0.0665**
Aftern.	**<0.0001**	**<0.0001**	**<0.0001**	ns	**<0.0001**	ns	**0.0001**	**0.0005**	**0.0331**	ns	ns	ns
2015												
Morning	**<0.0001**	**0.0159**	ns	**0.0223**	**<0.0001**	ns	ns	ns	ns	ns	**0.0596**	ns
Midday	**<0.0001**	**0.0004**	**0.0359**	ns	**<0.0001**	ns	**0.0009**	**0.0096**	ns	ns	ns	ns
Aftern.	**<0.0001**	**0.0032**	**0.0046**	ns	**<0.0001**	**0.0214**	**0.0542**	ns	ns	ns	ns	ns
